# An ethnobotanical survey of edible fungi in Chuxiong City, Yunnan, China

**DOI:** 10.1186/s13002-018-0239-2

**Published:** 2018-06-15

**Authors:** Dongyang Liu, Hong Cheng, Rainer W. Bussmann, Zhiyong Guo, Bo Liu, Chunlin Long

**Affiliations:** 1 College of Life and Environmental Sciences, Minzu University of China, Beijing, 100081 China; 2 Saving Knowledge, Casilla, 13092 La Paz, Bolivia; 3 Institute of Botany, Chinese Academy of Sciences, Beijing, 100093 China; 4 Kunming Institute of Botany, Chinese Academy of Sciences, Kunming, 650201 China

**Keywords:** Edible fungi, Market, Production chain, Sustainability, Ethnobotany

## Abstract

**Background:**

Chuxiong, known as “the City of Fungi,” is rich in fungal resources and traditional knowledge related to fungal biodiversity. The local environment is an excellent habitat for a wide variety of edible fungi. In addition, the region is home to many ethnic minorities and especially the Yi ethnic group who has a long history for traditionally using fungi as food or medicine. The aims of this review are to provide up-to-date information on the knowledge about, and traditional management of, fungi in this area and give advice on future utilization and conservation.

**Methods:**

Field surveys and in-depth semi-structured interviews were used to gather data. Ethnomycological data was collected from 67 informants in the summer of 2015.

**Results:**

Twenty-two edible fungal species were recorded both as food or non-timber forest products (NTFPs), used to increase income, and the importance of this resource for the Yi ethnic group was evaluated.

**Conclusion:**

Abundant and diverse wild genetic resources and a large production chain of edible fungi were recorded in Chuxiong. However, because of over-harvesting, the wild edible fungi are facing increasing threats. Suggestions are proposed to allow sustainable use of fungi resources, including (1) promotion of diversification of transportation, (2) development of fungi cultivation to improve quality and supply and reduce harvest pressure, (3) improvement of public awareness for environmental protection and sustainable development, and (4) promotion of eco-tourism and development of fungi catering in rural agro- and slow-food tourism.

## Background

Yunnan Province is located in the southwest part of China. This province harbors more than 15,000 plant species and is often called the “Kingdom of Plants” [1]. It has a wide variety of forest and soil types due to its unique natural mountainous environment [2, 3], which also creates unique microclimates and ideal conditions for a wide variety of edible fungi. The edible fungi in Yunnan are well-known in the world, and production is large and widespread. Yunnan is one of the richest sources of edible fungi in China, which in itself has more than 600 species of edible fungi, 30% of the edible fungi species in the world [4]. The annual production amounts to about 50,000 t, which does not include fungi from cultivation [5–7]. The production of edible fungi in Yunnan is the second largest agricultural export activity in the region. As a special industry, production of edible fungi is one of the important economic sources of the mountain areas [8]. With the growing emphasis on the conservation of biodiversity, the status and importance of the industry and fungi as a source for livelihoods become more and more prominent. Currently, there are about 15 million farmers working with edible fungi, and more than 400 factories process edible fungi, with more than 20 million people directly employed in the industry. Edible fungi in Yunnan are exported to 20 countries and regions, with Japan, the European Union, and the USA being the biggest traditional markets. South America, Africa, Russia, and ASEAN are the emerging markets [9]. The markets also show local differences, with, for example, *Tricholoma matsutake* (Chinese: 松茸; pinyin: song rong) mainly exported to Japan and *Boletus* (Chinese: 牛肝菌; pinyin: niu gan jun) and *Morchella esculenta* mainly exported to European countries.

The use and industrial production of edible fungi are ubiquitous in many areas around the globe, with distinct local characteristics. There are other regions rich in traditions of using edible fungi, such as Svaneti in Georgia, where 67 species of edible fungi were reported [10]. People living in Palas Valley, northeast Pakistan, like to eat *Morchella esculenta* (L.) Pers. (Chinese: 羊肚菌; pinyin: yang du jun) and use it as a part of their income source [11]. In Poland, 32 species of fungi were sold by local people in open-air markets [12]. There also are wild edible fungi found in other areas of China, but those areas do yet not count on an industrial production chain like we found in Chuxiong, Yunnan. Twenty-two fungi species were reported from Tewo County, Gansu, China, where people used to sell and store dried fungi [13]. In contrast, in Shaanxi, central China, few fungi species are traditionally used by local people, and production is not organized [14]. Only five taxa of edible fungi were recorded in Zhouqu County, Gansu, China. These edible fungi are however not harvested for commercial use [15]. Wild edible fungal resource development in Chuxiong relies mainly on collecting without paying and free trade. Most unemployed farmers in Chuxiong will take their children during summer vacation in July and August each year to pick wild edible fungi. During our survey in 2015, each household will earn around 1500 USD in this period, which provides positive social and financial benefits.

At present, China’s edible fungi output ranks first in the world, and it has become an important industry in China [16]. However, due to the high economic value of fungi, over-harvesting is very common [17–19]. A lack of unified management leads to environmental degeneration, and wild edible fungal resources cannot get effective protection. Currently, production decreases year after year [20]. *Tricholoma matsutake*, *Ophiocordyceps sinensis* (Chinese: 冬虫夏草; pinyin: dong chong xia cao), *Termitomyces albuminosus* (Chinese: 鸡枞菌; pinyin: ji zong jun), and other famous wild edible fungi are gradually getting endangered in Chuxiong and other areas [21–23]. Therefore, wild collection needs to be combined with artificial cultivation based on the unique environment and climate conditions in Yunnan [24]. Moreover, yield and quality of edible fungi need to be improved, and local wild edible fungal resources need to be managed sustainably. The main aims of this study were (1) to record wild edible fungi species sold in the traditional markets of Chuxiong, (2) to gain insights into the local edible fungi industry chain, (3) to document associated ethnobotanical knowledge, and (4) to give some suggestions about the development of a more sustainable edible fungi industry chain and conservation of wild edible fungi.

## Methods

### Study area and Yi people in Chuxiong

**Fig. 1:**
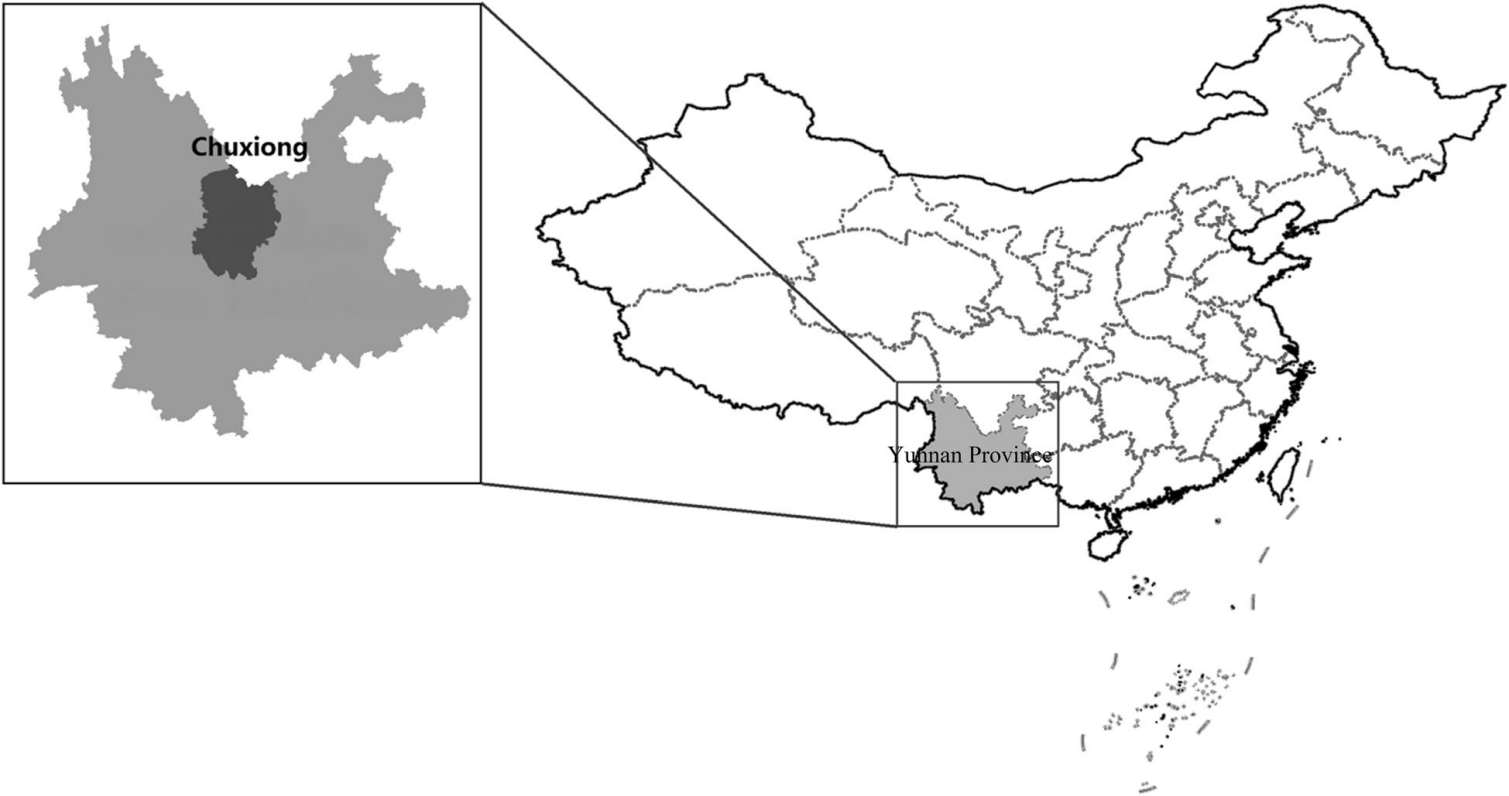
The location of Chuxiong Yi Autonomous Prefecture, Yunnan, China

Chuxiong City is located in the middle of Yunnan-Guizhou Plateau, west of Kunming, east of Dali, north of Pu’er, south of Panzhihua (Fig. 1). Its unique geographical location and climatic conditions create abundant plant resources. For example, wild edible fungi and wild herbs are very diverse in Chuxiong [25], with 540 species of fungi—90% of all fungal species in Yunnan. Because of the plant worship, Yi people in Chuxiong are conducting traditional management of fungal ecosystems, and the local fungal habitats are well conserved before. With a long history of eating wild fungi, the local Yi people have accumulated rich traditional knowledge. However, with the development of society and the impact of the mainstream culture, traditional culture is gradually losing. And the wild edible fungi are faced with over-harvesting [26, 27]. Especially in recent years, fungi became more well-known as a homology of medicine and food; some local people try to harvest more fungus and sell to merchants to earn money. Our research team visited Chuxiong City to collect information on edible fungi, including species available, edible value [28–31], medicinal value [32], and market conditions, to gain in-depth knowledge about edible fungi and their trade.

With a population of 8.7 million, Yi is the sixth largest minority group in China and mainly distributed in Yunnan, Sichuan, and Guizhou Provinces and Guangxi autonomous region. They mainly inhabit mountainous areas or river valleys. Yunnan Province holds the largest population of the Yi nationality, around 4.06 million; the Yi ethnic group is one of the most complex groups with many branches with different traditional costume, culture, language, constructions, etc. [33].

Some scholars believe that the Yi ethnic group was formed by ancient Qiang people in northwest China around 6000–7000 years ago. At first, they were separated into six tribes due to different living conditions: Wu (Chinese: 武), Za (Chinese: 乍), Nuo (Chinese: 糯), Heng (Chinese: 恒), Bu (Chinese: 布), and Mu (Chinese: 慕), and then, they migrated to southwest China. Six major tribes settled down in different directions. Their languages evolved into six major language groups, and each language group has 30–50 dialects. People belonging to different major language groups cannot communicate with each other. And even people who are identified as one branch of Yi with same family names, after 7–11 generations, traditionally, they will hold a special event “Naimu (Chinese: 耐姆)” to give different families new family names, and this ceremony boosts the diversification of ethnic branch.

Chuxiong Yi Autonomous Prefecture is the largest residence of the Yi nationality and the largest Yi Autonomous Prefecture in the country. Due to historical and geographical factors, Chuxiong has two Yi branches: Wu and Za. And Yi people in Chuxiong call themselves Nousu (Chinese: 诺苏), Nasu (Chinese: 纳苏), and Niesu (Chinese: 聂苏). The study population was chosen from them. The Yi language belongs to the Yi Branch of the Tibeto-Burman Group of the Sino-Tibetan Language Family. It is divided into the Northern, Eastern, Southern, Southeastern, Western, and Central dialects now. Each dialect is also divided into many local dialects, and it is very difficult to communicate with each other using different dialects [34–36]. The interviewed Yi people in Chuxiong speak Central Yi dialects [37].

As base for our study, we consulted the literature to assess the current situation of local edible fungi and the growing season of local wild edible fungi [38–41]. Fieldwork was conducted in summers of 2008 and 2015. The surveys were carried out in three counties of Chuxiong Prefecture: Wuding, Nanhua counties, and Chuxiong City. We investigated six different sizes of wild edible fungi markets in Chuxiong, and the large markets include “wild edible fungi wholesale market of Nanhua, Chuxiong”, “Chuxiong wholesale market of center agricultural products” and “wild edible fungi trading center of Kangzhao, Wuding.” The edible wild fungi trading center in Wuding is the most representative market, which is surrounded by numerous Yi villages (such as Xiaofakuai, Dafakuai, Limi, Jijiezi, and Fawo). These Yi villages are surrounded by mountains. Because of the moderate climate, plentiful rainfall, and fertile soil, these mountains (such as Taishan, Daheishan, Wozhangshan, Qiaoshan, Jidanshan, and Luoshan) are suitable for the growth of wild edible wild fungi. During the harvest period of wild edible fungi, unemployed farmers who pick fungi would have trade at the market. These wild edible fungi would be purchased by local Yi people or exported to other places.

### Ethnomycological research methods

Semi-structured interviews were carried out with local people in order to document the fungal species, edible value, medicinal value, and market transactions. The interview locations include villagers’ homes, edible fungi markets (including individual fixed purchase and food market), pine forest, and dehydrated edible fungi processing factory. Chuxiong is surrounded by mountains, and it is a long distance between countries because of continuous mountain roads. It is necessary to find local Yi guides to lead us to the interview locations. A total of 67 informants including 40 men and 27 women, all from Yi ethnic groups, with a large majority of middle-aged and younger people, were interviewed. Key informants included local guides, drivers, and five people collecting edible fungi as their career. All interviews were conducted after obtaining the oral prior informed consent of the individual participants. Usually Tibetans and Yi have the tradition of collecting mushrooms, and Yi people consume more mushroom, so most of the sellers and local customers are Yi people; of course, many overseas customers can get fresh mustache and other mushrooms in their own country.

Each survey documented the local name, frequency of use, and other values of the edible fungi held by informants. To understand the local wild edible fungi conditions and environment, we also assessed the local market share of various edible fungi, in order to make better recommendations for a more sustainable use of the resource. We collected 2–3 specimens for each species, total of 51 specimens, which were deposited at Minzu University of China (Beijing) and Kunming Institute of Botany herbaria. The specimens were later verified by the local Yi people in the market, authors, plant taxonomists, and experts from the authors’ institution.

The collected ethnobotanical data of edible fungi included scientific name, local name, and information about the production chain. Scientific name, local name in Chuxiong, habitat characteristics, main chemical components, and nutritional and medicinal value were recorded for each edible fungi species in Table 1.

**Table 1:** Fungal species found and their usages

Scientific name	Vernacular name (pinyin/Chinese/Yi)	Habitat characteristics	Main chemical components	Nutritional and medicinal value	Accession number of specimens
*Agaricus silvicola* (Vittad) Peck. (Agaricaceae)	san juer 伞菌	Forest, grow alone or in a small group		Edible, tasty, but there are some cases of poisoning. Harvest time: summer, autumn	s.n.
*Lactarius deliciosus* (L.) Gray (Agaricaceae)	mao cao jun 茅草菌	Grow in pine forest	Rich in proteins, crude fiber, unsaturated fatty acids, nucleic acid derivatives, vitamins B1, B2, vitamin C [45]	Strengthen body, condition the stomach, relieve pain, nourish lungs, and regulate breath; anti-cancer. Harvest time: summer	Y-M-070
*Vascellum pratense* (Pers.) Kreisel (Agaricaceae)	ma pi pao 马皮泡	Grow alone, in grassy areas, forest edges		Edible when they are young. Harvest time: spring, autumn	s.n.
*Sarcodon imbricatus* (L.) P. Karst. (Bankeraceae)	hu zhang jun 虎掌菌	Grow in forest with humic soil		Condition the stomach, nourish lungs, and regulate breath; anti-cancer. Harvest time: summer	Y-M-069
*Boletus luridus* Schaeff*.* (Boletaceae)	jian shou qing 见手青	Forest edges, grow alone or in a small group	Rich in proteins, amino acids, minerals, and polysaccharides [46]	Harvest time: summer	Y-M-068
*Boletus edulis* Rostk. (Boletaceae) [47]	mei wei niu gan jun 美味牛肝菌 Yi: ba hmu	Broad-leaved forest, grow alone or in a small group	Rich in polysaccharides, alkaloids (mainly of choline), putrescine, sterols [48]	Alleviate cold symptoms [49]. Harvest time: summer	Y-M-058
*Cantharellus cibarius* Fr. (Cantharellaceae)	huang si jun 黄丝菌 Yi: va cy hmu	Grow in forest of north temperate zone [50]	Rich in proteins, fats, carbohydrates, vitamins, carotene, crude fiber, phosphorus, and a variety of mineral nutrients [51]	Clean liver, improve vision, diuresis, nourish lungs, and regulate breath. Harvest time: summer	Y-M-062
*Ophiocordyceps sinensis* (Berk.) G.H. Sung et al. (Clavicipitaceae)	chong cao 虫草 Yi: se ge bbup ddi		Rich in mannitol, SOD, and polysaccharides	Anti-cancer, anti-tumor, nourish lungs, and regulate breath. Harvest time: spring	Y-M-064
*Ramaria botrytis* (Pers.) Ricken (Gomphaceae)	shua ba jun 刷把菌	Forest, grow alone		Edible, delicious taste, nourish the stomach, anti-cancer. Harvest time: summer, autumn	s.n.
*Ramaria madagascariensis* (Henn.) Corner (Gomphaceae)	sao ba jun 扫把菌	Coniferous forest, grow alone or in a small group		Edible, delicious taste. Harvest time: summer, autumn	s.n.
*Helvella atra* Oeder (Helvellaceae)	pi tiao jun 皮条菌	Grow clustered or scattered in forests		Prevent pernicious anemia, improve neurasthenia, cholesterol-lowering. Harvest time: summer, autumn	s.n.
*Hericium erinaceus* (Bull.) Pers*.* (Helvellaceae)	yang mao jun 羊毛菌 Yi: nyut o hmu	Grow on the top of trees	Rich in unsaturated fatty acids	Anti-ulcer, anti-inflammatory, anti-tumor, protect liver, strengthen immunity, anti-aging, and enhance blood circulation. Harvest time: spring, summer, autumn	Y-M-061
*Termitomyces albuminosus* (Berk.) Heim (Lyophyllaceae)	ji zong jun 鸡枞菌	Have symbiotic relationship with termite nests, grow in broad-leaved and coniferous forest	Rich in proteins, crude fat, crude fiber, and a variety of mineral nutrients [52]	Improve immune system, improve sleep. Harvest time: summer, autumn	Y-M-060
*Termitomyces microcarpus* (Berk et Brome) R. Heim (Lyophyllaceae)	huo ba ji zong 火把鸡枞	Have symbiotic relationship with termite nests, grow in groups	Rich in proteins [53]	Protect stomach. Harvest time: summer, autumn	s.n.
*Morchella esculenta* (L.) Pers. (Morocellaceae) [54]	yang du jun 羊肚菌 Yi: yo qy tie hmu	Grow in coniferous forests and broad-leaved forests	Rich in vitamin B1, B2, B12 [55]	Protect stomach, nourish the lungs, and strengthen immunity. Harvest time: spring, summer, autumn	Y-M-067
*Phallus indusiatus* Vent. (Phallaceae)	zhu gu niang 竹姑娘	Grow in hot and humid areas	Rich in proteins, crude fat, and crude fiber [56]	Nourish lungs and regulate breath, improve sleep, strengthen immunity. Harvest time: autumn	Y-M-066
*Lactarius volemus* (Fr.) Fr. (Russulaceae)	nai jiang jun 奶浆菌	Grow in conifer mixed forest	Rich in a variety of essential amino acids, protein, fat, vitamins, and a variety of mineral nutrients [57]	Anti-cancer, lower blood pressure, protect liver, detoxifying, diuresis. Harvest time: spring, summer, autumn	s.n.
*Russula virescens* (Schaeff.) Fr. (Russulaceae) [58]	qing tou jun 青头菌	Grow in mixed forest		Protect liver, protect eyesight. Harvest time: summer, autumn	s.n.
*Russula nitida* (Pers.) Fr. (Russulaceae) [59]	xiao hong jun 小红菌 Yi: zzit hmu	Grow in broad-leaved forest, solitary or in groups		Harvest time: summer, autumn	s.n.
*Sparassia crispa* (Wulfen) Fr. (Sparassidaceae)	hua er xiu qiu jun 花饵绣球菌	Grow in mountains, pine forest, central and western of Yunnan		Anti-aging, cholesterol-lowering, anti-cancer, strengthen immunity. Harvest time: summer	Y-M-059
*Stropharia rugosoannulata* Farl. ex Murrill (Sparassidaceae)	zhou huan qiu gai gu 皱环球盖菇	Grow in forest edges, grassy areas, gardens, garbage dumps, sawdust pile, cattle dunghill	Rich in vitamins and a variety of mineral nutrients [60]	Nourish brain. Harvest time: spring, summer, autumn	s.n.
*Tricholoma matsutake* (S. Ito et S. Imai) Singer (Tricholomataceae)	song rong 松茸 Yi: te hmu	Grow in natural forests without any pollution and human intervention	Rich in proteins, vitamins, and a variety of mineral nutrients [61]	Strengthen immunity, anti-cancer, anti-aging, prevent cardiovascular disease, improve gastrointestinal function, protect liver. Harvest time: summer	Y-M-063

## Results and discussion

**Fig. 2:**
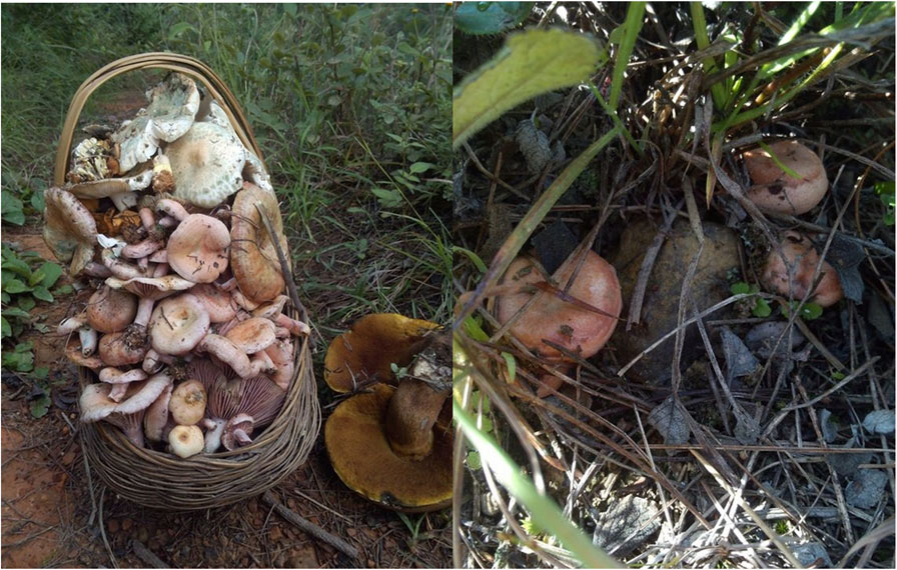
Field collection of fungi in Chuxiong

Our study found 22 species of edible fungi, which are most gathered from markets and a few collected from the wild (Fig. 2). These edible fungi are also mentioned by participants. These species were most frequently used by local people in summer and autumn. Most interviewees (39 villagers) said they had sold wild edible fungi to a purchasing company. This could increase their income but also increase the consumption of wild edible fungi, and over-harvesting might cause ecological damage. Some species mentioned in literature we did not encounter in the surveys.

Chinese name, pinyin, and Yi names have been listed in Table 1. Among the 22 species investigated, most interviewees know around 18–20 species’ Chinese name and pinyin. Only 15 interviewees out of 67 know Yi names for 1–5 species. *Sparassia crispa* and *Stropharia rugosoannulata* are rarely seen. Although 50% of interviewers know they are edible, only seven of them know exactly the Chinese common names.

### The current status of wild fungi

Generally, wild fungi in Chuxiong mainly grow in coniferous forest, composed by *Pinus yunnanensis* and *Pinus densata*, or mixed forests. Different vegetation types and different geographical environments result in a wide diversity of wild fungi.

In our investigation, the most common edible fungi were *Boletus edulis* Rostk., *Termitomyces albuminosus* (Berk.) Heim, *Morchella esculenta* (L.) Pers., *Phallus indusiatus* Vent., and *Tricholoma matsutake* (S. Ito et S. Imai) Singer. They were largely supplied to domestic and foreign markets.

According to interviews, a household could earn around 1500 USD by selling mushrooms during the harvest period, which improves the living standards of the local people. However, the mushroom resources are limited, and the local villagers did often collect indiscriminately. For example, when they found small *Termitomyces albuminosus* (Berk.) Heim, they would cover it with pine needles. Then, they would to dig it when it grew and protect the mycelium so that the fungi could continue to grow. This ensured both the development of ecological sustainability and the sustainability of their income.

Because more and more factories were built in recent years, demanding more prime material, some sensitive fungi species are decreasing. Old people indicated there were a lot of *Boletus edulis* Rostk. before, but now, the villagers indicated the need to climb to higher altitudes to find the resource. Wild *Tricholoma matsutake* (S. Ito et S. Imai) Singer, one of the most famous edible fungi in the world, is becoming very scarce and hard to find in the field. It is thus essential to protect the environment to also protect the local industry chain.

In Nanhua County, the “Mushroom Food Culture Festival” is held every year and has been held for 14 sessions so far. Nanhua County proved to be especially rich for mushroom diversity, in our research.

This county is located in the western part of the Chuxiong Yi Autonomous Prefecture, in a high elevation area. The climate is dominated by the subtropical monsoon. The local environment is highly suitable for fungi. *Tricholoma matsutake* exports account for a large share of production, and “Nanhua *Tricholoma matsutake*” is protected by the National Protection of Geographical Indications. The annual income from wild edible fungi amounted to 7677 t, valued at 80 million USD.

### Conservation of fungi based on traditional management of Yi people

The local Yi ethnic group in Chuxiong have over time developed a profound knowledge for utilizing edible fungi and developed their own management of ecosystem. Local people traditionally use edible fungi for food, medicine, religious worship, and culture, and Chuxiong conserves the highest fungal diversity in Yunnan. The reason could be attributed to the Yi minorities maintaining their customs of the utilization and management of local fungi diversity [42]. It is thus very important to utilize indigenous cultural knowledge for ecological and environmental conservation. We learned from the interviews that local people harvest wild edible fungi without destroying their hyphae. However, nowadays, with increasing loss of traditional culture, young harvesters prefer to simply uproot the fungi. For that reason, we suggest that the indigenous resource management knowledge and culture diversity should be conserved and extensively studied.

Chuxiong is becoming more and more commercialized, and the famous rich fungi resources and unique Yi traditional culture have become a famous destination for eco-tourists. This creates a complex relationship between traditional and modern management.

### Edible fungi industry chain

**Fig. 3:**
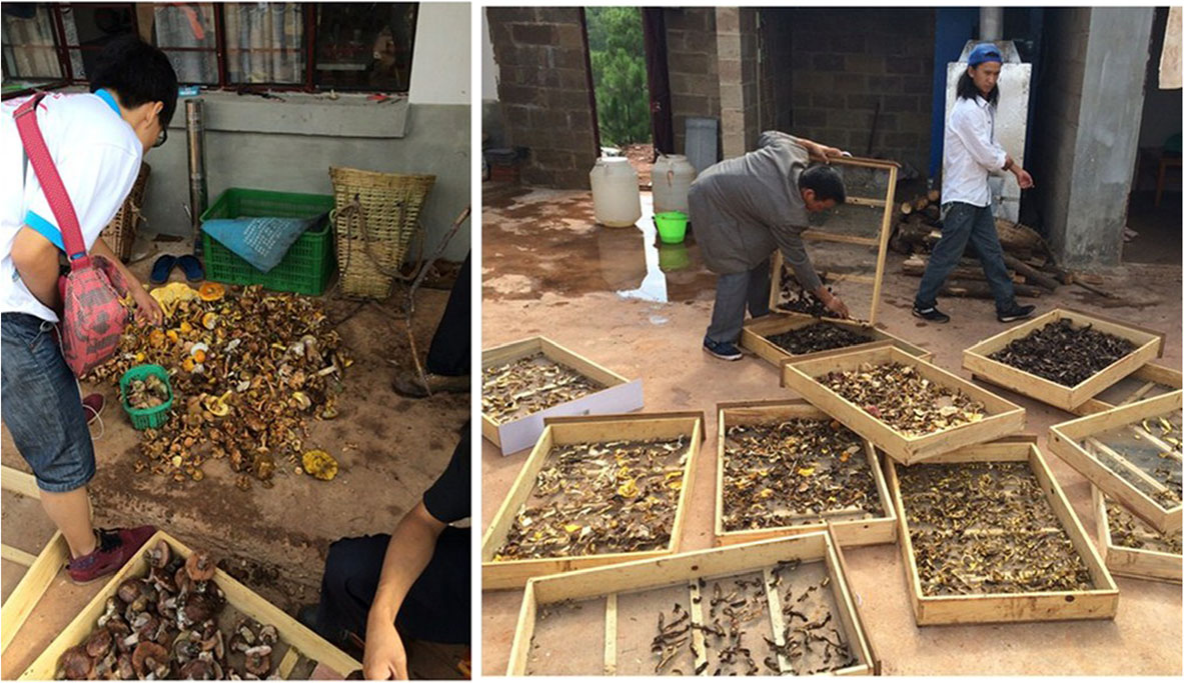
The processing of dried edible fungi

**Fig. 4:**
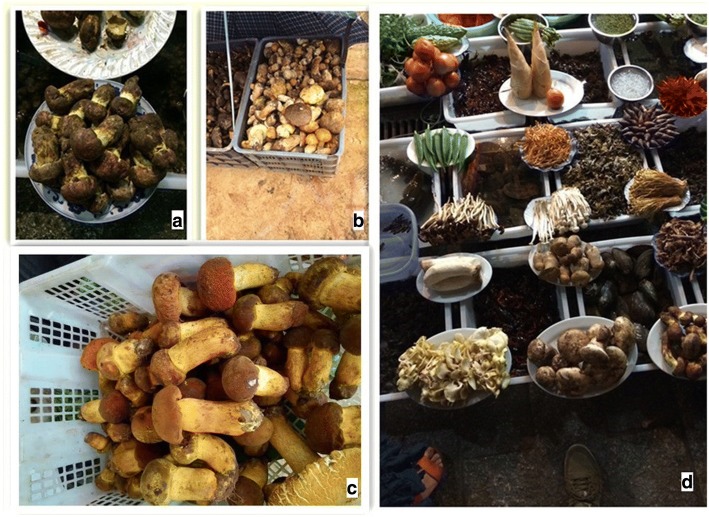
**a**, **b** The most common edible fungus: *Boletus edulis* Rostk. (Chinese: 美味牛肝菌; pinyin: mei wei niu gan jun). **c** The most common edible fungus: *Boletus calopus* Fr. (Chinese: 美柄牛肝菌; pinyin: mei bing niu gan jun). **d** A variety of edible fungi shown in a restaurant

In our research region, factories that produce dried edible fungi were generally built on hillsides, close to collectors’ villages (Figs. 3 and 4). This has several advantages: (1) land is cheap, (2) it is convenient to purchase fresh edible fungi from the market when close to the village, and (3) it is convenient to transport when close to the road.

**Fig. 5:**
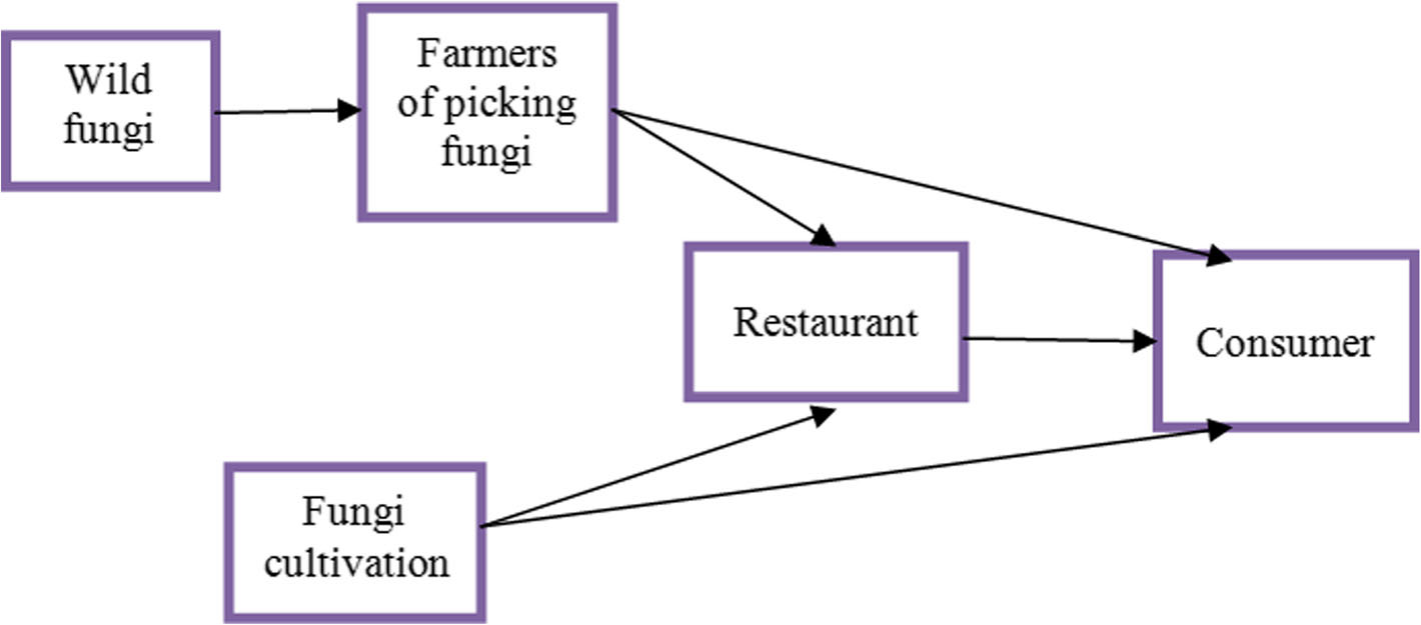
The main production chain of edible fungi

**Fig. 6:**
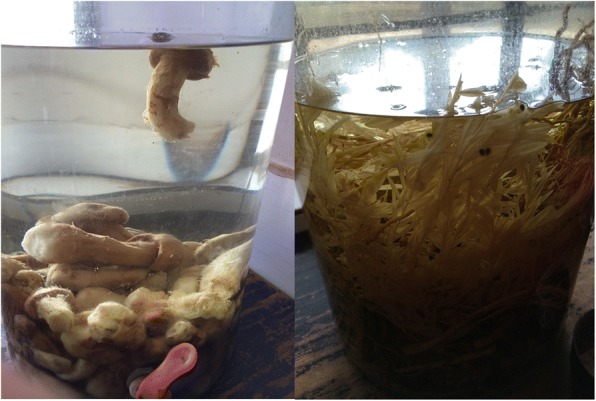
*Ophiocordyceps sinensis* (Berk.) G.H. Sung et al. and *Tricholoma matsutake* (S. Ito et S. Imai) Singer steeped in liquor

During our visit, we also investigated the local wild fungi production chain (Fig. 5). The *Tricholoma matsutake* industry, as example, allows to illustrate all aspects of the fungal production and trade. The Yunnan *Tricholoma matsutake* industry chain is composed of farmers, middlemen, manufacturers, and distributors (Fig. 6). The upstream production chain comprises mostly farmers, producing, breeding, picking, and primary packaging the resource. The midstream are middlemen, linking farmers to manufacturers and distributors. The number of middlemen is directly related to the transaction costs and efficiency. Downstream production includes manufacturers and distributors.

The Yunnan *Tricholoma matsutake* production chain is short and resource-based. The resource appears over-exploited and growingly endangered, threatening mountain farmers’ income. To develop the Yunnan *Tricholoma matsutake* production chain under a concept of sustainability is of paramount importance.

The key factor in the development of the production chain of Yunnan *Tricholoma matsutake* is supply capacity and consumer demand. The demand for Yunnan *Tricholoma matsutake* is increasing, and the quality of the product, as well as supply capacity, has become a priority. However, the farmers, due to scattered and small-scale production, provide relatively low-quality material. The lack of the ability to control the market and other factors becomes a bottleneck of product supply. Industrial management through enterprise groups tries to unite the large number of scattered farmers to achieve continuous supply. Moreover, collectivization, and large-scale development, can closely link all aspects of the production chain and promote the flow of technology, capital, and other factors to extend the production chain. Members of this production group found that the Yunnan *Tricholoma matsutake* production chain is currently in its infancy, characterized by low-tech production and the lack of strong competitiveness. Therefore, we recommended to use the power of government departments to improve the industrial management of Yunnan wild edible fungi. Local government should increase financial input and establish industrial cooperation organization systems to create an even wider market according to the scientific planning. Because the resources of wild edible fungi are limited, local government should encourage more famers to engage in the cultivation of edible fungi to ensure the sustainable development [43].

In Beijing, we found *Termitomyces albuminosus* (Berk.) Heim and *Boletus edulis* Rostk. made into snacks and sold in the large supermarkets. *Morchella esculenta* (L.) Pers., *Phallus indusiatus* Vent. and *Tricholoma matsutake* (S. Ito et S. Lmai) Singer are dried for sale. Some fungi, such as fresh *Cantharellus cibarius* Fr. and *Helvella atra* Oeder are however not easily preserved and thus difficult to produce in an industrial chain.

## Conclusions

**Fig. 7:**
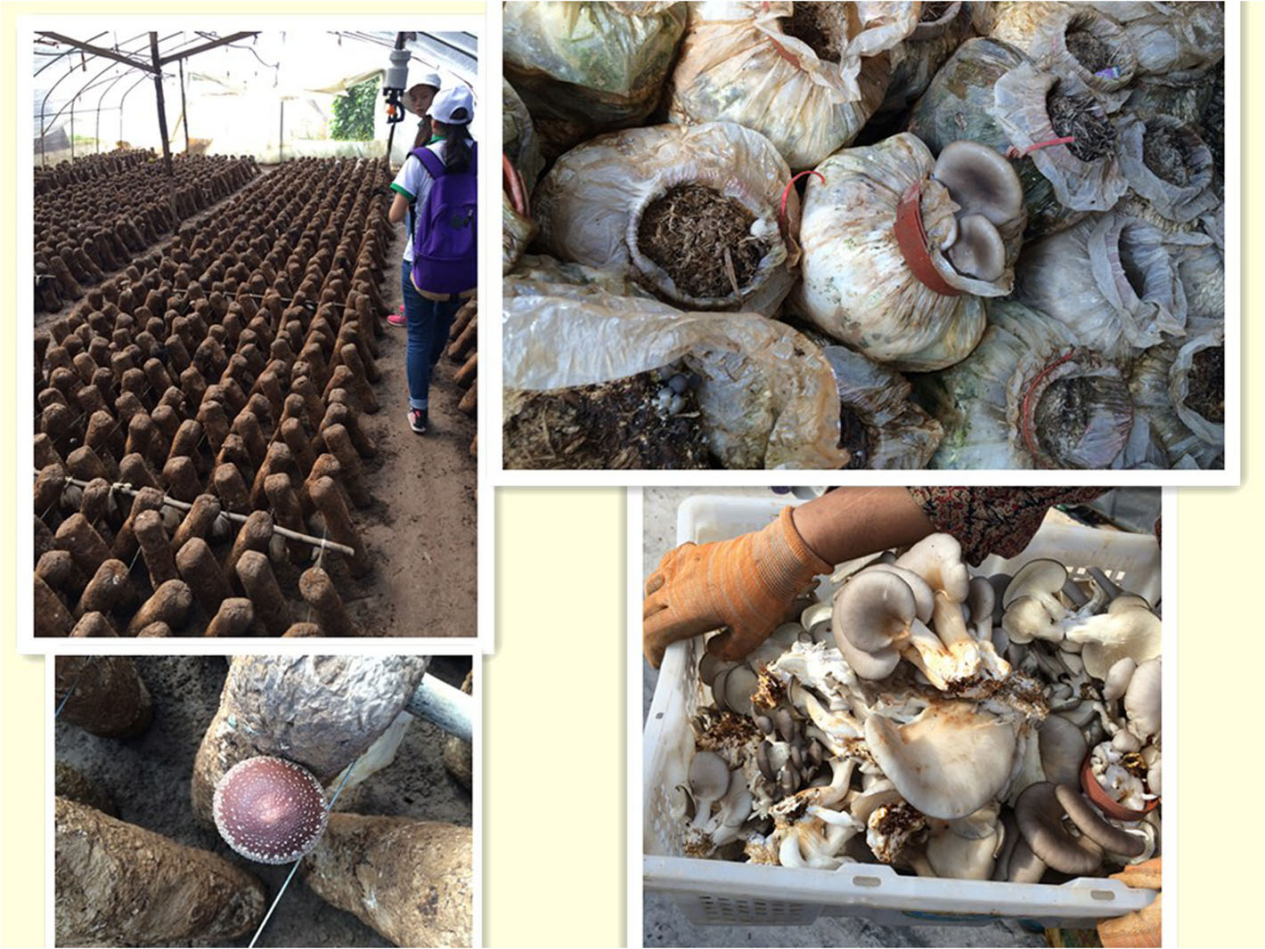
Industrial production of fungi

Our study provides ethnomycological data on the wild edible fungi in Chuxiong, Yunnan. Twenty-two wild edible fungi are found used for traditional food by Yi people, which are more than other countries in China but less than Tshopo Province in the Democratic Republic of the Congo [13–15, 44]. However, the commercialization of wild edible fungi in Chuxiong is more advanced because of the support by government, including holding the “Mushroom Food Culture Festival” and building the well-developed road network. Wild edible fungi are traditional food for the Yi people. Due to economic development, wild edible fungi have become much commercialized, and while the fungal industry chain has boosted the local economy, it has also created some unexpected social and ecological problems. Due to the medicinal value of wild edible fungi, local people in Chuxiong even used *Ophiocordyceps sinensis* (Berk.) G.H. Sung et al. and *Tricholoma matsutake* (S. Ito et S. Imai) Singer to be steeped in liquor (Fig. 6). Through this investigation, we propose the following suggestions for promoting local edible fungi development and rural development:

1. Promote the diversification of transportation. Due to easy spoilage, edible fungi are normally transported dry, rather than fresh. The development of air transport systems has been approved by the Yunnan government, which would be helpful expand the markets of edible fungi. Also, it can reduce the loss in transit.
2. Develop cultivation of fungi to improve quality and supply and reduce harvest pressure (Fig. 7).
3. Improve the public awareness of environmental protection and promote sustainable development. Over-exploitation is detrimental to economic development and threatens the survival and development of future generations.
4. Promote eco-tourism and develop fungi catering in rural agro- and slow-food tourism. Edible fungi are very popular, but awareness about possible threats and conservation issues is limited. It is necessary to build brands, if the government wants to develop the edible fungi industry in the future.
